# Integration with Transcriptomic and Metabolomic Analyses Reveals the *In Vitro* Cytotoxic Mechanisms of Chinese Poplar Propolis by Triggering the Glucose Metabolism in Human Hepatocellular Carcinoma Cells

**DOI:** 10.3390/nu15204329

**Published:** 2023-10-11

**Authors:** Yuyang Guo, Zhengxin Liu, Qian Wu, Zongze Li, Jialin Yang, Hongzhuan Xuan

**Affiliations:** 1 School of Life Science, Liaocheng University, Liaocheng 252059, China; guoyuyang1026@163.com (Y.G.); 2210150205@stu.lcu.edu.cn (Z.L.); 2110150205@stu.lcu.edu.cn (Q.W.); 2210150201@stu.lcu.edu.cn (Z.L.); 2 College of Life Science, Shihezi University, Shihezi 832000, China; 3 Yili Prefecture Agricultural and Rural Bureau, Yili 835000, China

**Keywords:** Chinese poplar propolis, hepatocellular carcinoma cells, cell metabolism, transcriptome sequencing, metabolomics

## Abstract

Natural products serve as a valuable reservoir of anticancer agents. Chinese poplar propolis (CP) has exhibited remarkable antitumor activities, yet its precise mechanisms of action remain elusive. This study aims to elucidate the *in vitro* cytotoxic mechanisms of CP in human hepatocellular carcinoma cells (HepG2) through comprehensive transcriptomic and metabolomic analyses. Our evidence suggested that CP possesses a great potential to inhibit the proliferation of HepG2 cells by targeting the glucose metabolism. Notably, CP exhibited a dose- and time-dependent reduction in the viability of HepG2 cells. Transcriptome sequencing unveiled significant alterations in the cellular metabolism, particularly within glucose metabolism pathways. CP effectively restrained glucose consumption and lactic acid production. Moreover, the CP treatment led to a substantial decrease in the mRNA expression levels of key glucose transporters (*GLUT1* and *GLUT3*) and glycolytic enzymes (*LDHA*, *HK2*, *PKM2*, and *PFK*). Correspondingly, CP suppressed some key protein levels. Cellular metabolomic analysis demonstrated a marked reduction in intermediary products of glucose metabolism, specifically fructose 1,6-bisphosphate and acetyl-CoA, following CP administration. Finally, key compounds in CP were screened, and apigenin, pinobanksin, pinocembrin, and galangin were identified as potential active agents against glycolysis. It indicates that the effectiveness of propolis in inhibiting liver cancer is the result of the combined action of several components. These findings underscore the potential therapeutic value of propolis in the treatment of liver cancer by targeting glycolytic pathways.

## 1. Introduction

Malignant tumors pose a serious threat to human health, with liver cancer ranking as the third most common cause of cancer-related deaths worldwide [1]. Infections with the Hepatitis B and C viruses are major risk factors for the development of hepatocellular carcinoma. Nonalcoholic steatohepatitis linked with chronic illnesses has become a more prominent risk factor for liver cancer in the Western world in recent years [2]. Targeted therapeutic drugs for liver cancer include sorafenib, regorafenib, sunitinib, erlotinib, gefitinib, and bevacizumab. These drugs can target specific molecules or signaling pathways in cancer cells to inhibit their growth, infiltration, and metastasis [3]. However, these targeted chemotherapeutic drugs are highly toxic and prone to drug-resistant phenotypes. Therefore, it is important to search for anticancer drugs in natural products and analyze their mechanisms of action for cancer treatment.

Propolis is a resinous substance collected by honeybees (*Apis mellifera* L.) by mixing the exudates from plant resin [4]. The chemical composition of propolis is highly complex and variable due to the raw materials that are collected by the honeybees. There are different types of propolis around the world, including green Brazilian propolis (mainly from *Baccharis dracunculifolia*), European propolis (*Populus nigra* L.), red Brazilian propolis (*Dalbergia ecastophyllum*), Russian propolis (*Betula verrucosa Ehrh*), and Cuban and Venezuelan red propolis (*Clusia* spp.) [4]. More than 600 compositions have been identified from different types of propolis [4,5]. Chinese propolis mainly belongs to the “*Populus*” propolis, which predominantly contains phenolic acids and flavonoids [5]. Propolis has a broad range of biological activities, such as antiviral, antibacterial, antioxidant, anti-inflammatory, immunomodulatory, and antitumor activities, etc. [6,7,8]. The antitumor effects of propolis have attracted increasing attention from researchers in recent years, with studies indicating that Chinese propolis in particular has excellent antitumor activity; however, its mechanisms of action are not yet fully understood [9,10,11].

Abnormal metabolism is a hallmark of cancer, and tumor cells are metabolically reprogrammed to meet the energy and biomolecules required for malignant proliferation and metastasis [12]. The Warburg effect has been demonstrated as a marker of energy metabolism in several types of tumor cells [12,13,14]. Aerobic glycolysis in cells regulates their metabolism to ensure an adequate supply of ATP, facilitating the adaptation of cells to altered survival conditions and their rapid proliferation. This enhances the tolerance of tumor cells to harsh conditions and increases their growth [13]. The glycolytic capacity of cancer cells is 20–30 times higher than that of normal cells, and this high rate of glycolysis leads to the rapid production of ATP, while lactate dehydrogenase (LDH) converts the majority of the generated pyruvate into lactate in the cytoplasm [15]. These acidic products change the tumor microenvironment, which is conducive to tumor invasion and infiltration, as well as the inhibition of the activity and function of tumor immune effector cells [15]. Therefore, targeting the aerobic glycolysis of tumor cells can selectively inhibit tumor cell proliferation and development.

Aerobic-glycolysis-related transport proteins and key enzymes are involved in cell development. An increasing number of studies identified a significant upregulation of the expression of glucose transporter proteins (GLUTs) in tumor cells, particularly GLUT1 and GLUT3 overexpression in a variety of solid tumors. When glucose crosses the cell membrane into the cytoplasm, it undergoes phosphorylation to form glucose-6-phosphate, which initiates glycolysis. The activity and protein expression levels of key glycolytic enzymes, such as lactate dehydrogenase A (LDHA), hexokinase II (HK2), M2 pyruvate kinase (PKM2), and phosphofructokinase (PFK), are significantly upregulated in cells [16,17]. The inhibition of GLUT and key glycolytic enzymes of the glucose metabolism by small-molecule inhibitors or RNAi can slow the growth of many tumor cells [17]. Propolis has excellent antitumor activity, but the effects of propolis on the cell metabolism, especially glycolysis and gluconeogenesis in liver cancer cells, remain unknown. 

In the present study, based on transcriptomics and cell metabolomics, we explored these effects to further elucidate the antitumor mechanisms of propolis.

## 2. Materials and Methods

### 2.1. Chemicals and Reagents

Dulbecco’s modified Eagle medium (DMEM), fetal bovine serum (FBS), penicillin/streptomycin (10,000 U/mL), and phosphate buffer solution (PBS) were purchased from Gibco (Pittsburgh, PA, USA). The primer was synthesized by Sangon Biotechnology Co., Ltd. (Beijing, China). Primary antibodies against GAPDH and β-actin were obtained Santa Cruz Biotechnology (Santa Cruz, CA, USA). Primary antibodies against LDHA, HK2, and PKM2 were purchased from Cell Signaling Technology (Beverly, MA, USA). The protease and phosphatase inhibitors were from ABclonal biotech (Shanghai, China). A CCK8 kit was purchased from Dojindo Laboratories (Kumamoto, Japan), while the BCA protein assay and SDS-PAGE gel configuration kits were from Beyotime Biotechnology (Shanghai, China). Ethanol absolute (≥99.5%) was purchased from Shanghai Macklin Biochemical Co., Ltd. (Shanghai, China). Formic acid (88%), acetonitrile (99.95%), and methanol (99.9%) were purchased from Fisher Chemical (Pittsburgh, PA, USA).

### 2.2. Preparation of the Chinese Poplar Propolis Ethanol Extract

Propolis was collected from Nanyang of Henan Province in North China in 2017 (voucher specimen no. CP17110702), and poplar (*Populus* spp.) was the main plant source. The extraction method of propolis was consistent with one of our previous studies [9]. In brief, propolis was frozen, milled, and extracted using 99.5% ethanol (*v*/*v*), and then subjected to ultrasonication at 40 °C for 3 h. The supernatant from three extractions was combined, filtered, and concentrated under reduced pressure to a constant weight and stored at −20 °C. The major chemical compositions of the Chinese poplar propolis (CP) were analyzed using an ultra-high-performance liquid chromatography/quadrupole time-of-flight mass spectrometry system in a negative ion mode.

### 2.3. UHPLC/Q-TOF-MS

The CP and cellular metabolite samples were analyzed using UHPLC/Q-TOF-MS (6545, Agilent Technologies, Beijing, China). The UHPLC conditions were as follows: The column was a ZORBAX Eclipse Plus C18 column (2.1 × 100 mm, 1.8 μm); the column temperature was 30 °C. Mobile phase A was 0.1% formic acid in water and B was 0.1% formic acid in acetonitrile; the flow rate was 0.3 mL/min. The injection volume was 2 μL, and the gradient elution program was carried out according to the following procedure: 0 min/5% B, 2 min/5% B, 20 min/100% B, and 25 min/100% B with a postruntime of 5 min. Mass spectrometry was conducted using an electrospray ionization (ESI) ion source. The samples were detected under positive/negative ion conditions, respectively. The nebulizer voltage was 35 psi, the dry gas (N2) temperature was 325 °C, and the flow rate was 10 L/min. The capillary voltage was 3500 V, the sheath gas temperature was 370 °C, and the flow rate was 12 L/min. The fragmentor voltage was 135 V, and mass spectra were obtained in a mass range of 100–1700 *m*/*z* [18].

Agilent Profinder 8.0 (Agilent Technologies, Beijing, China) was used to extract and correct the peak area, mass number, and retention time of the original metabolomics data (including primary and secondary mass spectrometry data), which were exported in .cef format. The data in .cef format were then imported into Mass Profiler Professional 15.1 for statistical analysis. ID Browser10.0 (Agilent Technologies, Beijing, China) was used to identify all compounds for subsequent metabolic pathway analysis. MetaboAnalyst 5.0 was used to import the names of potential cellular metabolites into an online system for metabolite enrichment analysis.

### 2.4. Cell Culture

The human liver cancer cell line HepG2 was generously gifted from the Institute of Apiculture, Chinese Academy of Agricultural Sciences. HepG2 cells were cultured in DMEM supplemented with 10% (*v*/*v*) FBS, and the culture medium contained 100 μg/mL of streptomycin and 100 U/mL of penicillin. The cells were cultured in a humidified environment containing 5% CO_2_ at 37 °C. When the cells reached 70% confluence, they were collected for subsequent experiments.

### 2.5. Cell Viability Assay

HepG2 cells were seeded into 96-well cell culture plates at a density of 5 × 10^4^/mL. When the cells became 70% confluent, they were treated with CP (25, 50, or 100 μg/mL) for 24 and 48 h, respectively. A CCK-8 kit was used to determine cell viability.

### 2.6. Quantitative Real-Time Reverse-Transcriptase Polymerase Chain Reaction

The total RNA of cells was extracted using TRIzol reagent (Invitrogen, CA, USA). A Nanodrop 2000 spectrophotometer (Thermo Fisher Scientific, Waltham, MA, USA) was used to determine the concentration of the extracted RNA. Then, 1000 ng of total RNA was used as a template for cDNA synthesis, and a PrimeScript RT kit (TaKaRa, Dalian, China) was used for the reverse transcription process. Fluorescent quantitative real-time PCR (qRT-PCR) was performed using the QuantStudio™ 1 Real-Time fluorescent quantitative PCR system (Applied Biosystems, Carlsbad, CA, USA) and TB Green^®^ Premix Ex Taq™ II kit (TaKaRa, Dalian, China) as instructed by the reagent vendor. The 2^−ΔΔCt^ method was used to analyze the data, normalized to the expression of the housekeeping gene (*β-actin*). The primer design is shown in Table S1.

### 2.7. RNA Sequencing

Total messenger RNA (mRNA) was extracted from the propolis-treated or control HepG2 cells. Total RNA samples with an RNA integrity number (RIN) > 7.0 and a 28S:18S ratio ≥ 1.5, which were used for subsequent library preparation and sequencing, were obtained from CapitalBio Technology (Beijing, China). High-throughput RNA sequencing (RNA-seq) was performed using the Illumina NovaSeq sequencer (Illumina, San Diego, CA). Sequencing quality was assessed using FastQC (v0.11.9) (https://www.bioinformatics.babraham.ac.uk/projects/fastqc/, accessed on 26 October 2020) to filter out low-quality data. Clean reads were then compared to the reference genome using Bowtie2 [19]. Using HISAT2 (v2.1.0) software, the processed reads from each sample were aligned to the public reference genome. Transcript abundances were measured as fragments per kilobase of transcript per million fragments mapped (FPKM) using HTSeq [20]. The DESeq2 package was used to analyze the differentially expressed genes (DEGs) between groups. The differential gene screening criterion was: |log_2_FC| ≥ 1 (FC: multiple of differential expression). Bioinformatics analysis was subsequently performed using oebiotech (https://cloud.oebiotech.cn/, accessed on 29 May 2023). A *p*-value of 0.05 was chosen as the cut-off criterion.

### 2.8. Measurement of Glucose Consumption and Lactate Production

After 24 and 48 h of CP intervention, the cell culture medium was collected, centrifuged at 1000 rpm for 10 min, and the supernatant was taken. Lactate and glucose levels in the culture medium were determined using a lactate test kit and a glucose test kit (Nanjing Jiancheng Bioengineering Institute, Nanjing, China) according to the manufacturer’s instructions.

### 2.9. Western Blotting

Whole-cell lysates were prepared as previously described [9]. Briefly, the cells were lysed with RIPA lysis solution (containing 1% protease inhibitor and 1% phosphatase inhibitor) on ice for 30 min following two washes with ice-cold PBS, and then scraped and centrifuged at 13,000 rpm for 8 min at 4 °C. Then, the supernatant was transferred to a new centrifuge tube, and the protein concentration was determined using a BCA kit (Beyotime Biotechnology, Shanghai, China). The protein was denatured by proportionally adding 5 × SDS and heating it at 100 °C for 5 min. A total of 30 μg of protein was electrophoresed using 8–12% sodium dodecyl sulfate-PAGE (SDS-PAGE) and transferred onto polyvinylidene fluoride (PVDF) membranes. The membrane was blocked with 5% skimmed milk powder for 1 h at 25 °C and then incubated with primary antibody overnight at 4 °C. The membrane was washed three times with 1 × PBST to remove the unbound primary antibody and incubated with a secondary antibody coupled with horseradish–peroxidase (diluted to a ratio of 1:2000–10,000) for 1 h at 25 °C. After washing the membrane two times with 1 × PBST, the membrane was incubated with ECL substrate and detected using Amersham Image600 (General Electric, Norwalk, CT, USA).

### 2.10. Cellular Metabolite Extraction

The HepG2 cells were treated with 100 μg/mL of CP for 48 h when they were confluent to 70% in the six-well plates, and the control group was replaced with fresh medium. The cell culture medium was discarded, and the plates were washed three times with prechilled 1 × PBS, then 1 mL of ice-cold 80% methanol (*v*/*v*) was added to each well on ice and extracted at −80 °C for 1 h. The cells were scraped into the centrifuge tubes with a cell scraper, and 1 mL of 80% methanol (*v*/*v*) was added to each well again to rinse and try to transfer all cells; cells from three wells were combined into one centrifuge tube. The experiment was repeated six times. The supernatant was centrifuged at 10,000 rpm for 30 min at 4 °C and blow-dried with nitrogen. The cellular extracts were redissolved with 80% methanol (*v*/*v*). After centrifugation at 15,000 rpm for 20 min at 4 °C, the supernatant was added to the injection vial for UHPLC/Q-TOF-MS measurement. The quality control (QC) process consisted of an equal amount of each sample and was used to check the instrument stability and calibrate all samples [21].

### 2.11. Molecular Docking Analysis

We previously discovered that seven compounds, including apigenin, chrysin, galangin, caffeic acid phenethyl ester (CAPE), caffeic acid benzyl ester, pinocembrin, and pinobanksin, showed significant inhibitory activity on a number of cancer cells, including HepG2, and hence picked these seven compounds for molecular docking [9,22]. Molecular docking used the protein–small molecule automated docking software AutoDock vina (v1.1.2); the more stable the conformation and the lower the binding energy, the higher the likelihood of the interaction. In detail, the ligand structures of the seven components of propolis were downloaded from the Pubchem database (https://pubchem.ncbi.nlm.nih.gov/, accessed on 23 August 2023) and the three-dimensional structures of the target proteins were downloaded from the RCSB-PDB database (https://www.rcsb.org/, accessed on 23 August 2023). Then, water molecules were removed, hydrogen bonds were formed, and excess ligands were eliminated from the proteins. Visualization of the results was performed via PyMol [23,24,25].

### 2.12. Statistical Analysis

Data were subjected to one-way ANOVA using GraphPad Prism software (version 8.0) and IBM SPSS software (version 26.0). Each experiment was repeated at least three times. Data are expressed as the mean ± S.E.M, with *p* < 0.05 representing a significant difference. RNAseq data were analyzed and visualized using the online platform, oebiotech. MetaboAnalyst 5.0 was used for visualization of the metabolomics data.

## 3. Results

### 3.1. Nontargeted UHPLC/Q-TOF-MS CP Analysis

The chemical composition of propolis is very complex and is strongly influenced by geographical location and plant origin. In previous studies, we isolated and identified 11 chemical components of propolis [9]. In order to gain a detailed understanding of the chemical composition of the CP used in the present study, we performed a compositional analysis of the sample in negative ion mode using ultra-high-performance liquid chromatography/Q-TOF-MS. Figure S1 shows the total ion current chromatogram (TIC). Supplementary Table S1 lists the TOP 100 compounds in CP.

### 3.2. CP Inhibited the Cell Viability of HepG2 Cells

**Figure 1:**
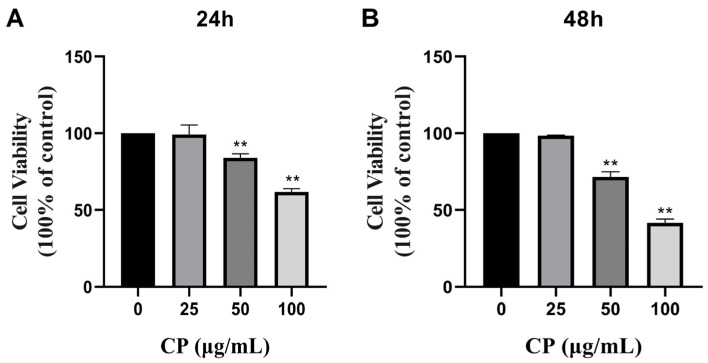
The effects of Chinese poplar propolis (CP) on the viability of HepG2 cells. (**A**,**B**) Cytotoxicity of CP (25, 50 and 100 μg/mL) in HepG2 cells at 24 and 48 h. (** *p* < 0.01 vs. control, n = 3.) Data are means ± S.E.M.

To investigate the inhibitory effect of CP on HepG2 cells, the HepG2 cells were treated with different concentrations of CP (25, 50, or 100 μg/mL) for 24 and 48 h, followed by the detection of cell viability using a CCK-8 kit. The cell viability of the HepG2 cells significantly decreased in a dose- and time-dependent manner after CP treatment compared to the control group (Figure 1A,B).

### 3.3. Propolis Affected Metabolism-Related Transcriptome Alterations in HepG2 Cells

**Figure 2:**
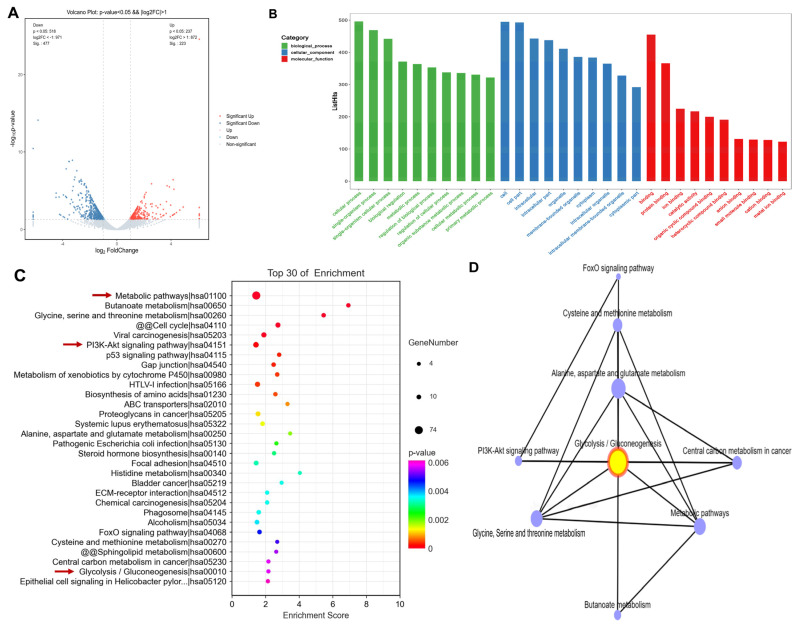
Transcriptomic analysis of HepG2 cells after CP treatment. (**A**) Volcano plot of DEGs in HepG2 for the propolis-treated and control groups. The *x*-axis and *y*-axis indicate log_2_ (fold change) and −log_10_ (*p*-value) of DEGs in HepG2, respectively. The red color represents upregulated genes, and the blue color represents downregulated genes. (**B**) GO pathway enrichment analysis of DEGs. The 30 most significantly enriched GO terms in a complex of biological process, molecular function, and cellular component branches are presented. (**C**) Scatter plots show the top 30 enriched KEGG pathways of DEGs. The rich factor is the ratio of DEGs numbers to all gene numbers annotated in this pathway term. A higher rich factor means a greater intensiveness. (**D**) KEGG network diagram. The TOP30 pathways were screened to obtain 8 pathways interconnected via glycolysis/gluconeogenesis.

Transcriptome sequencing was used to detect the differentially expressed genes in the propolis-treated cells. Additionally, the absolute value of logarithmic fold changes ≥ 1 and *t*-test *p*-values < 0.05 were taken as the thresholds for selecting DEGs. A total of 223 DEGs were upregulated and 477 DEGs were downregulated in the propolis-treated group compared to the control group; the results are shown Figure 2A. This indicates that the propolis-treated group was significantly different against the control group. These genes may be the target genes regulated by CP in HepG2 cells.

To gain insights into the function of these significantly altered DEGs, GO enrichment analysis was performed, especially at the biological process (BP), molecular function (MF), and cellular component (CC) levels. As shown in Figure 2B, the three most representative Gene Ontology (GO) terms in the CC domain were “cell”, “cell part”, and “intracellular.” The top three GO terms in the MF domain were “binding”, “protein binding”, and “ion binding”, while the five most enriched GO terms in the BP domain were “cellular process”, “single-organism process”, “single-organism cellular process biological regulation”, and “metabolic process”, suggesting that CP may affect the cellular metabolism of HepG2 cells. The cellular metabolic process is related to cancer cell survival.

To further understand the biological functions of the DEGs affected by CP, a KEGG pathway enrichment analysis was performed on the significantly altered DEGs. In Figure 2C, CP mainly affected the “metabolic pathways”, “butanoate metabolism”, “glycine, serine, and threonine metabolism” on HepG2. Interestingly, most of the enriched TOP30 pathways were related to the cellular metabolism, such as the central carbon metabolism, amino acid metabolism, and glycolysis. The results of the KEGG analysis are consistent with those of the GO analysis. Moreover, glycolysis cross-talked several signaling pathways, such as “butanoate metabolism”, the “PI3K–AKT signaling pathway”, and the “FoxO signaling pathway” (Figure 2D). We previously found that propolis significantly downregulated the protein expression levels of the PI3K–AKT signaling pathway in HepG2 cells [9]. Therefore, the regulation of the HepG2 metabolic pathway, especially the glycolytic pathway, allowed CP to exert an anticancer effect.

### 3.4. CP Suppressed Glucose Consumption and Lactate Production

**Figure 3:**
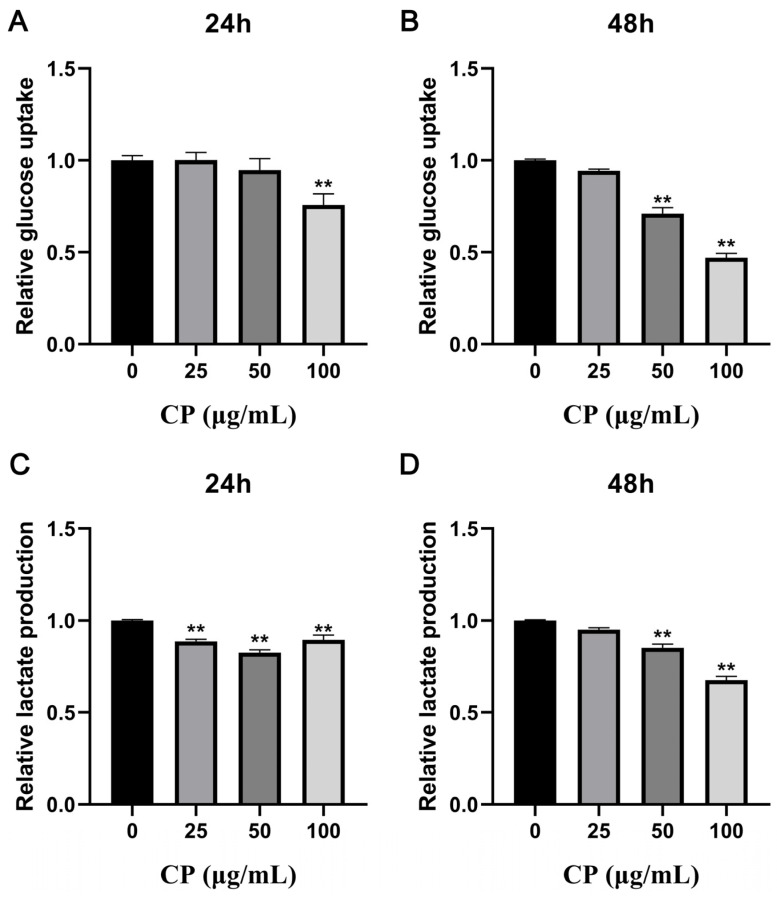
Analysis of glucose consumption and lactate production. The supernatant was collected after 24 and 48 h of CP treatment for glucose and lactate determination. (**A**,**B**) Relative glucose consumption at 24 and 48 h of CP intervention at different concentrations. (**C**,**D**) Relative lactate production at 24 and 48 h of CP intervention at different concentrations. Normalized to control group. ** *p* < 0.01, n = 3.

Generally, normal cells ingest glucose and undergo glycolysis under anaerobic conditions to produce lactate, but tumor cells also metabolize glucose under aerobic conditions via the glycolysis pathway, which is known as the Warburg effect. Therefore, we investigated the effects of CP treatment on glucose uptake and lactate production. As shown in Figure 3A,B, CP intervention significantly inhibited the glucose uptake (*p* < 0.01). In particular, 100 μg/mL CP treatment for 48 h could reduce glucose uptake by more than 50%. CP also reduced lactate production to varying degrees (*p* < 0.01) and maintained an inhibitory effect for 48 h (Figure 3C,D).

### 3.5. CP Inhibited the mRNA Expression of Glucose Transporter Proteins GLUT1 and GLUT3

**Figure 4:**
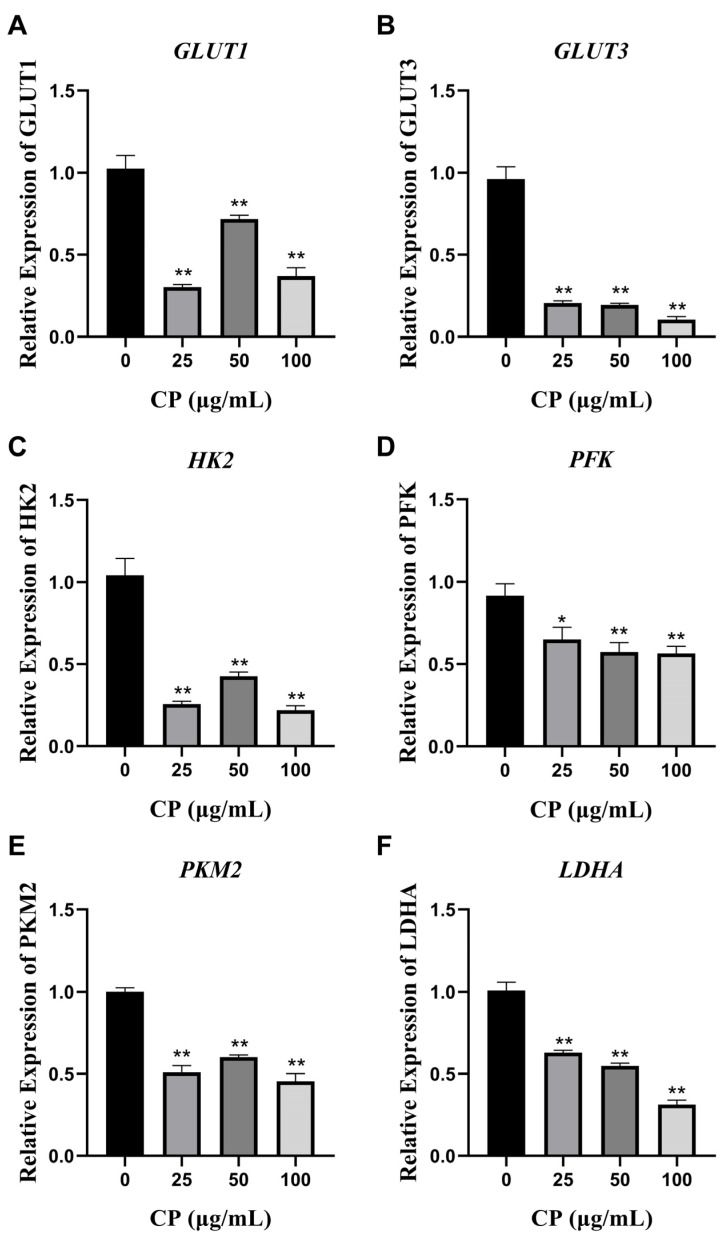
Gene expression analysis. Effects of CP on the mRNA expression of genes related to glucose transporters and glycolytic rate-limiting enzymes in HepG2 cells. Cells were treated with CP (25, 50, 100 μg/mL) for 36 h or 20 min. The mRNA levels of *GLUT1* (**A**), *GLUT3* (**B**), *HK2* (**C**), *PFK* (**D**), *PKM2* (**E**), and *LDHA* (**F**) were quantified using qRT-PCR and normalized to *β-actin*; the levels of gene expression in the control group were set to 1. The data shown represent means ± SEMs values from three independent experiments. Individual groups were compared using ANOVA (* *p* < 0.05, ** *p* < 0.01 compared with the control group, n = 3).

Glucose is a polar molecule that cannot directly cross cell membranes; therefore, glucose transporters play an important role in glucose uptake in cells. The qRT-PCR results show that CP significantly inhibited the gene levels of the glucose transporter proteins GLUT1 and GLUT3 (Figure 4A,B).

### 3.6. CP Suppressed the Levels of Four Glycolytic Key Enzymes

**Figure 5:**
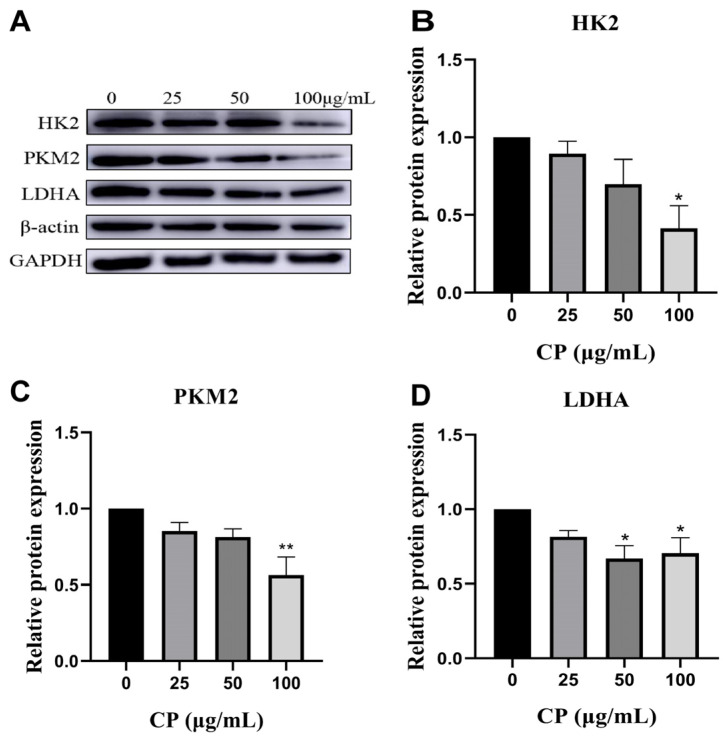
Protein expression analysis. (**A**) Expressions of HK2, PKM2, and LDHA in HepG2 cells at 48 h. (**B**–**D**) Quantification of relative protein expressions of HK2, PKM2, and LDHA in HepG2 at 48 h. Data are means ± S.E.M. * *p* < 0.05, ** *p* < 0.01 vs. control, n = 3. The blots of relative proteins had been cropped during the experiment.

Extracellular glucose enters the cell via GLUTs and undergoes phosphorylation to form glucose-6-phosphate (G6P), initiating glycolysis. As shown in Figure 4C,E,F, CP treatment for 36 h significantly reduced the mRNA levels of essential glycolytic enzymes, such as HK2, PKM2, and LDHA. We also observed a significant inhibition of the protein expression levels of these three key enzymes following CP treatment (Figure 5A–D). Since the time of gene expression was unknown, no change in PFK gene expression was detected after CP treatment for 36 h; therefore, after a 20 min CP intervention, we detected PFK gene expression. CP treatment for 20 min significantly decreased the levels of PFK mRNA expression (Figure 4D).

### 3.7. Differential Cellular Metabolite Analysis

**Figure 6:**
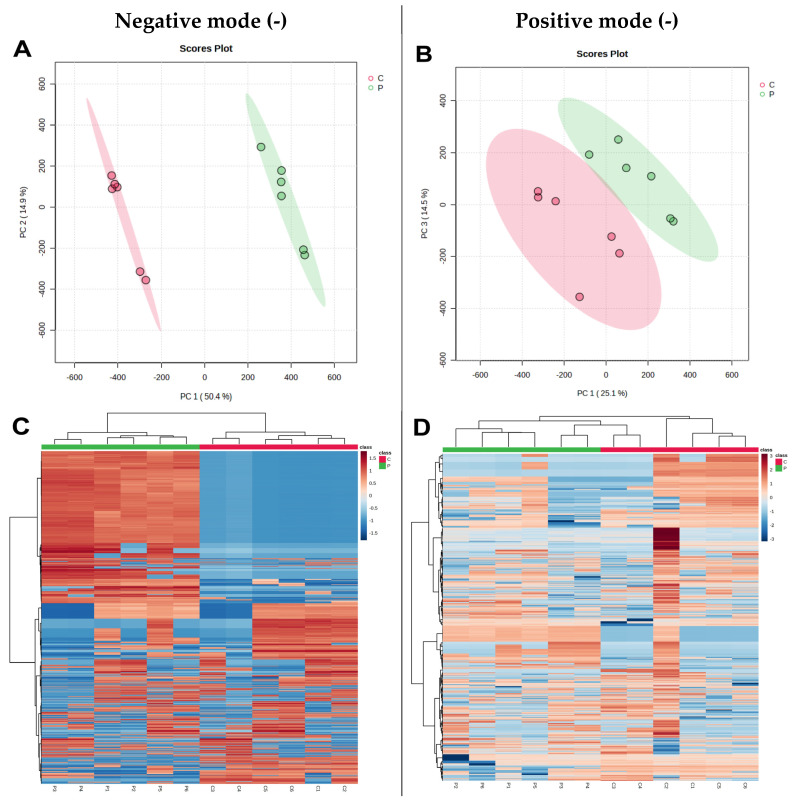

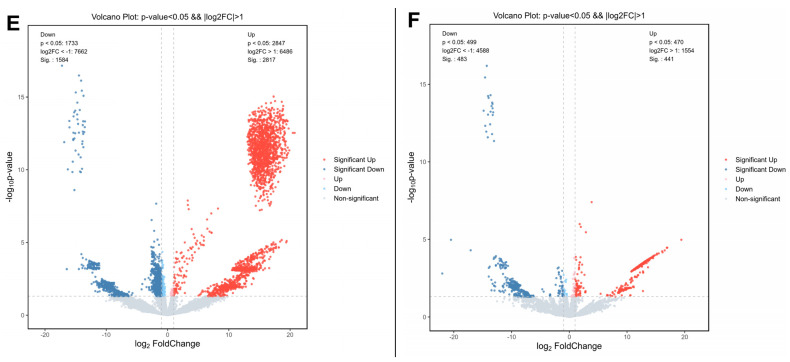
Multivariate statistical analyses of metabolites in HepG2 cells (PCA). (**A**,**B**) The PCA score plots in negative and positive modes, respectively. (**C**,**D**) Cluster heatmap analysis of metabolites at CP and control exposure. (**E**,**F**) Volcano plots of the metabolic in control and CP treatment groups. (C: control, P: Chinese poplar propolis).

Based on gene and protein expression results, we hypothesized that CP could affect changes in the metabolites of HepG2 cells. Therefore, we analyzed the metabolite changes in CP-treated and control cells using a UPLC-Q-TOF-MS-based metabolomics approach in negative/positive ion modes, respectively. The MetaboaAnalyst 5.0 (https://www.metaboanalyst.ca/, accessed on 30 May 2023) online analysis platform for metabolic data was used to compare the differentially metabolized compounds between the CP and control groups. The overall distribution between samples was observed using unsupervised PCA. After CP treatment, PCA showed a clear classification of the metabolite composition of each group, with samples from the same group being clustered together. The PC1 variable in PCA explained 50.4% of the variation in the original data in the negative mode. The principal component variable PC1 in the positive mode could explain 25.1% of the variation in the original data (Figure 6A,B), which showed significant differences between the control and CP-treated groups, suggesting that CP causes significant changes in the metabolites within cells. Volcano plots and cluster heatmaps were used to determine the differences in the metabolic profiles between the different groups. CP had a noticeable effect on cellular metabolites. Metabolites were effectively separated in the control and CP groups (Figure 6C,D). The red areas in the cluster heatmaps indicate higher metabolite levels, while the blue areas represent lower metabolite levels. As shown in the volcano plots, there were more differential compounds between the CP-treated and control groups in the negative ion mode than in the positive ion mode (Figure 6E,F). In the *t*-test, *p* < 0.05 and |log_2_FC| > 1 were considered as criteria for discovering biologically significant different metabolites.

### 3.8. Analysis of the Differential Metabolites Associated with Glycolysis

**Figure 7:**
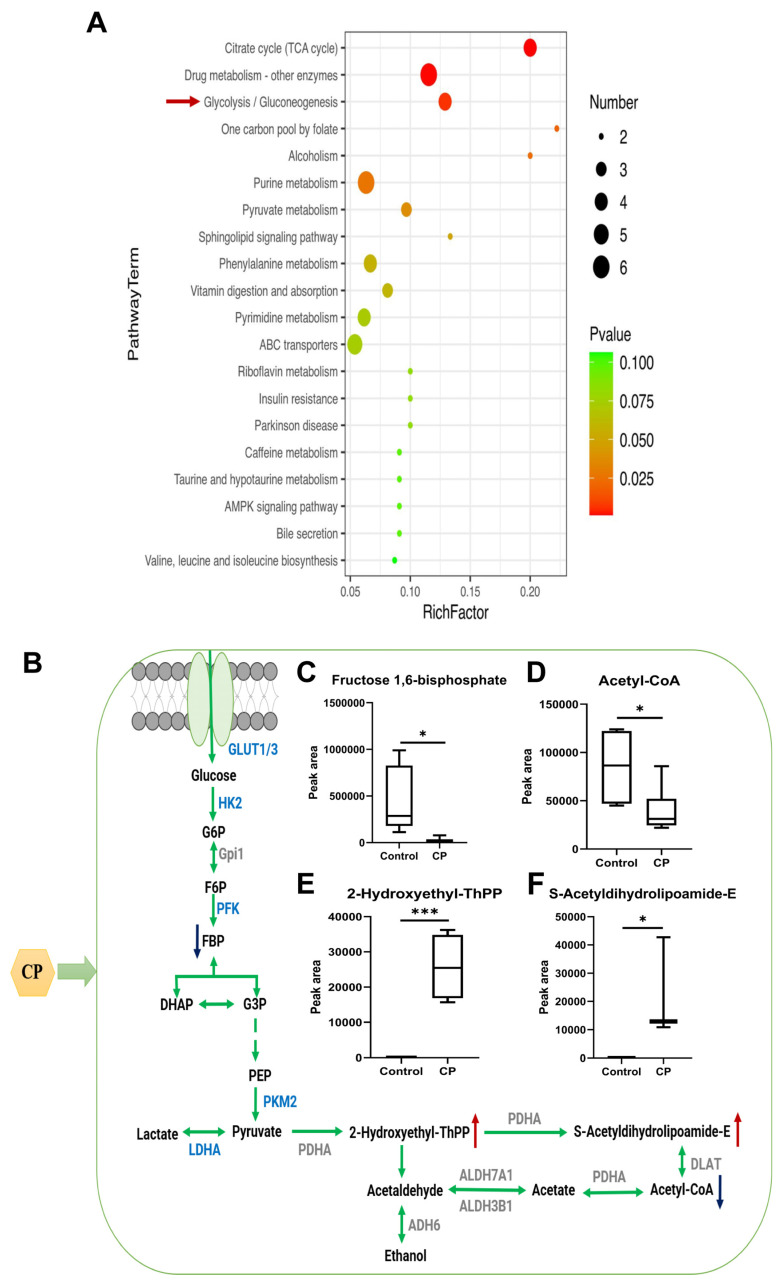
Effects of CP on metabolic pathways in HepG2 cells. (**A**) Analysis of metabolic pathway associated with the propolis anticancer effect using an enrichment analysis with an online OE Biotech. Bubble plots indicate the differential metabolic compound pathway enrichment analysis in the control and propolis-treated groups of HepG2. (**B**) Summary of the changes in glycolysis upon propolis treatment. Metabolites that decrease upon propolis treatment are indicated with blue arrows. The increased metabolites after propolis treatment are indicated by red arrows. Changes in identified metabolite biomarkers in response to the propolis treatment in the pathways of glycolysis/gluconeogenesis (**C**–**F**). Data for (**C**–**F**) are the means ± SEM of six replicates, * *p* < 0.05, *** *p* < 0.001 (in comparison to the control group via Student’s *t* tests, n = 6).

Based on the above criteria, the differential metabolites were screened and compared to the KEGG database to obtain metabolic pathway classification profiles (Figure 7A), and the three pathways with the highest enrichment were “citrate cycle (TCA cycle)”, “drug metabolism—other enzymes”, and “glycolysis/gluconeogenesis”. This indicates that the significant changes in the metabolites after CP treatment were highly correlated with glucose metabolism. In the glycolysis/gluconeogenesis metabolic pathway, the intermediate metabolites changed significantly after CP treatment, as shown in Figure 7B. The content of intermediate products, such as fructose 1,6-diphosphate (FBP) and acetyl-CoA, significantly decreased, and 2-hydroxyethyl-ThPP and S-acetyldihydrolipoamide-E levels were significantly upregulated (Figure 7C–F).

### 3.9. Molecular Docking Simulation of the Effects of Primary Active Components of Propolis on Glycolysis Key Enzymes

**Figure 8:**
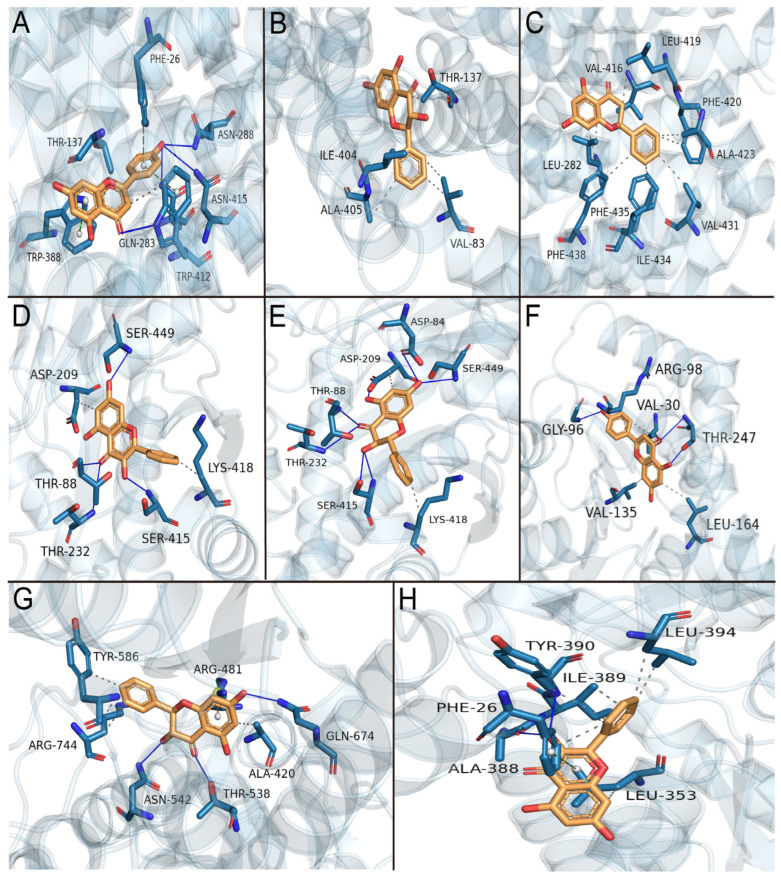
Combination of the active components of propolis and glycolysis key enzymes. The target protein structure is shown in blue and the brown represents the small-molecule ligand. (**A**) GLUT1–apigenin, (**B**) GLUT1–pinobanksin, (**C**) GLUT3–pinocembrin, (**D**) HK2–galangin, (**E**) HK2–pinobanksin, (**F**) LDHA–apigenin, (**G**) PFK–pinobanksin, and (**H**) PKM2–galangin. Blue line for hydrogen interactions, gray dashed line for hydrophobic interactions, light green dashed line for Π–Π stacking (P-type), dark green dashed line for Π–Π stacking (T-type).

According to the molecular docking data (Table 1), the binding energies of all the active components with proteins were less than −5 kcal·mol^−1^, indicating that the principal active compounds of propolis have a high affinity for proteins and good binding stability. GLUT1–apigenin, GLUT1–pinobanksin, GLUT3–pinocembrin, HK2–galangin, HK2–pinobanksin, LDHA–apigenin, PFK–pinobanksin, and PKM2–galangin were the best docking results of protein and propolis key active components, respectively. The main active components of propolis bind to proteins primarily through hydrogen bonding and hydrophobic interactions. Additionally, apigenin forms Π–Π stacking (T-type) and Π–Π stacking (P-type) interactions with GLUT1; formation of Π–cation interactions between pinobanksin and PFK; and galangin forms a Π–Π stacking (T-type) interaction with PKM2 (Table 2 and Figure 8).

## 4. Discussion

The altered cell metabolism caused by cancer has made it a popular target for the development of new therapies. Propolis has significant anticancer properties; flavonoids mainly exert an antitumor effect [26,27,28], but whether propolis affects the tumor cell metabolism remains unclear. Here, for the first time, we found that propolis interferes with the metabolism in hepatocellular carcinoma cells, especially the glycolysis pathway, inhibiting the expression of glucose transporter proteins and key glycolysis enzymes, downregulating intermediate metabolites, and ultimately inhibiting the malignant proliferation of liver cancer cells.

Recent studies have demonstrated that propolis and its components have the ability to inhibit various types of cancers, such as breast, colon, brain, skin, and blood cancers [29]. This study found that low doses of CP did not affect the viability and proliferation of HepG2 cells, but medium and high doses of CP showed a significant inhibitory effect. These results are consistent with previous research where the cell proliferation of HCC-LM3 cells was significantly inhibited after the intervention of high doses of chrysin, the main component of propolis [30].

Previous studies have shown that propolis can suppress the cell cycle, induce apoptosis, autophagy, and antiangiogenic activity, and modulate the tumor inflammatory microenvironment [31,32]. Additionally, propolis has been found to inhibit tumor cell proliferation through various signal pathways, including MAPK, PI3K/AKT/mTOR, JAK-STAT, TLR4-NF-κB, VEGF, and TGFβ [33]. However, these studies were not able to fully reveal the antitumor mechanisms of propolis. Propolis is a complex with multiple efficacy components, and its antitumor efficacy also reflects the characteristics of multiple pathways and targets. In the field of human health, transcriptome sequencing is gaining traction as a tool to identify therapeutic targets for diseases [34,35]. Transcriptome sequencing provides a more comprehensive understanding of the antitumor efficacy of propolis by revealing the genes and signaling pathways that propolis may regulate. A transcriptome analysis revealed that CP treatment resulted in significant changes in the expression of 700 mRNAs and exerted antihepatocarcinogenic effects, mainly by affecting ion binding and the cellular metabolism, especially the glucose metabolism.

Mammalian cells require an active energy metabolism to survive. Glucose monosaccharide enters the cell via the glucose transporter and initiates glycolysis as a significant source of cellular energy [36]. Lactate, a product of glycolysis, promotes stromal degradation, facilitates tumor metastasis, and evades immune surveillance [36]. Our results show that CP treatment significantly inhibits the production of lactic acid for the cellular consumption of glucose. This tentatively suggests that CP can inhibit aerobic glycolysis in hepatocellular carcinoma cells, and a similar phenomenon was observed in Licochalcone A-treated gastric cancer cells [37]. Next, we examined the effects of CP on GLUT1 and GLUT3 at transcriptional levels, showing that high concentrations of CP significantly inhibited the gene expressions of GLUT1/3. Quercetin, as a competitive inhibitor of GLUT1-mediated glucose uptake, is shown to have anticancer effects at elevated concentrations [38].

The development of HCC is highly correlated with the glucose metabolism. High glucose levels can cause reactive oxygen species (ROS) to build up, and surplus ROS can bind to DNA and cause the formation of HCC [39]. One-third of the genes involved in the glucose metabolism are continuously dysregulated, which is linked to the poor prognosis of HCC [40]. In normal cells, glycolysis converts one glucose molecule into two pyruvate molecules and generates ATP. Depending on the oxygen supply, pyruvate is either oxidized to carbon dioxide and water through the TCA cycle or, under anaerobic conditions, pyruvate produces lactate [41]. In contrast, tumor cells preferentially undergo glycolysis to produce lactate, even under aerobic conditions; this metabolic reprogramming is known as the Warburg effect. To produce energy quickly in cancer cells, glycolysis is preferred over the TCA cycle and oxidative phosphorylation (OXPHOS), and OXPHOS intermediates, including ATP, citrate, and ROS, negatively control three irreversible steps of glycolysis [42]. Thus, minimal dependence on OXPHOS is well suited in tumor cells [43,44,45]. Our previous studies show that tumor cells, including liver cancer cells treated with propolis, exhibit significantly higher levels of ROS, which may also be one of the reasons why propolis inhibits the glycolytic process [9]. The regulation of the key glycolytic enzymes in cancer cells is another important factor of propolis inhibiting tumor cell proliferation. Glycolysis is the first step of the glucose metabolism, consisting of ten enzymatic steps and three irreversible catalyzed reactions. HK2, PFK, and PKM2 catalyze three irreversible processes that are important regulatory points in the glycolytic process [46]. Enzyme activity can be altered in a variety of ways, such as inhibition brought on by an excess of metabolites, the phosphorylation of the enzyme, and transcription-induced changes in the enzyme concentration [46]. HK2 is overexpressed in a variety of tumor cells, and studies have shown that HK2 ablation delays lung and breast tumor progression [47], while HK2 silencing reinstates the flux of pyruvate to the TCA cycle and suppresses aerobic glycolysis, contributing to tumor therapy [48]. Licochalcone A reduces the levels of Akt in two different types of gastric cancer cells to inhibit HK2-mediated tumor glycolysis [37]. PFK has two isoforms, PFK1 and PFK2, both of which are post-translationally modified in cancer cells [49]. The knockdown of PFK2 in breast cancer cells accumulates ROS to induce apoptosis [50]. It has been demonstrated that PFK expression and activity are inhibited by epigallocatechin-3-gallate in hepatocellular cancer cells [51]. PK has four isoforms—PKM1, PKM2, PKL, and PKR. PKM2 is favored by tumor cells [52], making it a target for antitumor treatment [53]. Lactate dehydrogenase (LDH) catalyzes a reversible reaction, converting pyruvate into lactate, and the high-speed conversion of glucose to lactate results in elevated GLUTs in glucose uptake [54,55]. LDHA is overexpressed in a variety of tumor cells, and the silencing of RNAi causes apoptosis to occur in liver cancer due to increased ROS production [56]. Wogonin treatment inhibits the action of LDHA in human gastric cancer and lung adenocarcinoma cells [57]. Increased aerobic glycolysis is the hallmark of tumors. This study shows that propolis can target the glucose metabolism by decreasing the levels of glycolytic key enzymes, such as HK2, PFK, PKM2, and LDHA.

Among the multiple signaling pathways regulated by propolis, the modulation of metabolic pathways is the most significant. Based on metabolomics, principal component analysis plots showed a clear separation between sample points, suggesting that CP treatment had a greater effect on the metabolism of HepG2 cells. Additionally, altered tumor cell metabolism is a prominent feature of tumorigenesis and development [58]; thus, propolis might target the tumor cell metabolism to inhibit liver cancer cell proliferation. The results regarding cell metabolism further demonstrate that propolis modulates the tumor cell metabolism, including the citrate cycle, glycolysis/gluconeogenesis, one carbon metabolism, and the purine and pyrimidine metabolism. Under the intervention of CP, the content of intermediate products of glycolysis, such as FBP and acetyl-CoA, significantly decreased. In addition to the rapid production of ATP, intermediates of glycolysis are also precursors for the synthesis of many biomolecules, such as amino acids and lipids [46]. Cell proliferation involves the *de novo* synthesis of many biomolecules, and acetyl-CoA is a pivotal substance in the metabolism of energy substances in the body [59]. CP intervention leads to a reduction in acetyl-CoA levels, thereby inhibiting the synthesis of biomacromolecules, and thus cell proliferation.

The molecular docking results revealed that all seven propolis actives had a strong binding capacity with key glycolysis enzymes, with apigenin, pinobanksin, pinocembrin, and galangin being the best. Previous research found that all four flavonoids suppressed HepG2 cell survival [9]. Apigenin has been demonstrated to decrease GLUT1, HK2, PKM2, and LDHA production in tumor cells [60,61], and galangin has been shown to bind to the PKM2 site [62]. Combining these four flavonoids with glycolysis may give us with a fresh perspective on propolis’s antitumor activity, which can be examined further in future studies. Thus, CP is a polyphenol mixture with a broad range of targets, and its antitumor activity exceeds that of a single component, implying that propolis targets glycolysis for its anticancer efficacy as a result of the combined action of several components.

## 5. Conclusions

The distinguishing features of the tumor cell metabolism include increased glucose uptake and aerobic glycolysis, leading to the conversion of glucose into lactate. In our current investigation, by utilizing transcriptomics and metabolomics, we found that propolis has the potential to modulate the tumor cell metabolism, especially the glucose metabolism, by suppressing glucose consumption, glucose transporter proteins, glycolytic key enzymes, intermediate metabolites, and lactate production, ultimately resulting in inhibited cell proliferation. This could be due to the combined activity of several propolis components.

## Figures and Tables

**Table 1:** Docking binding energy of the main active components of propolis to protein molecules.

Protein Targets	PDB ID	Affinity/(kcal·mol^−1^)						
		Apigenin	Chrysin	Galangin	Caffeic Acid Phenethyl Ester	Caffeic Acid Benzyl Ester	Pinocembrin	Pinobanksin
GLUT1	6THA	−8.7	−8.6	−8.4	−8.5	−8.6	−8.6	−8.7
GLUT3	5C65	−6.2	−6.4	−6.4	−5.7	−6.0	−6.5	−6.4
HK2	2NZT	−7.4	−7.7	−7.8	−7.5	−7.5	−7.6	−7.8
LDHA	4JNK	−8.0	−7.6	−7.6	−7.0	−7.3	−7.6	−7.5
PFK	4XZ2	−7.4	−7.4	−7.6	−6.6	−6.9	−7.4	−7.7
PKM2	3GQY	−7.1	−7.3	−7.4	−7.3	−7.2	−7.2	−7.2

**Table 2:** Optimal results of molecular docking of the main active components of propolis with proteins.

	Apigenin–GLUT1	Pinobanksin–GLUT1	Pinocembrin–GLUT3	Galangin–HK2	Pinobanksin–HK2	Apigenin–LDHA	Pinobanksin–PFK	Galangin–PKM2
Affinity (kcal/mol)	−8.7	−8.7	−6.5	−7.8	−7.8	−8.0	−7.7	−7.4
Number of hydrogen interactions	4	-	-	4	7	3	3	2
Amino acid residues involved in hydrogen bonds	Gln283, Asn288, Trp412, Asn415	-	-	Thr88, Thr232, Ser415, Ser449	Asp84, Thr88, Asp209, Thr232, Ser415, Ser449	Gly96, Thr247	Thr538, Asn542, Gln674	Ala388, Tyr390
Number of hydrophobic interactions	2	5	10	2	2	4	4	7
Amino acid residues involved in hydrophobic interactions	Thr137, Trp412	Val83, Thr137, Ile404, Ala405	Leu282, Val416, Leu419, Phe420, Ala423, Val431, Ile434, Phe435, Phe438	Asp209, Lys418	Asp209, Lys418	Val30, Arg98, Val13, Leu164	Ala420, Tyr586, Arg744	Phe26, Leu353, Ile389, Tyr390, Leu394
Number of Π stacking	3	-	-	-	-	-	-	1
Amino acid residues involved in Π stacking	Phe26 (T), Trp412 (T), Trp388 (P)	-	-	-	-	-	-	Phe26 (T)
Amino acid residues involved in Π–cation interaction	-	-	-	-	-	-	Arg481	-

## Data Availability

The data used to support the findings of this study can be made available by the corresponding author upon request.

## Supplementary Materials

The following supporting information can be downloaded at: https://www.mdpi.com/article/10.3390/nu15204329/s1, Table S1: Sequences of the primers for qRT-PCR; Table S2: TOP 100 components in ethanol extract of propolis by HPLC-QTOF-MC; Figure S1. Total ion current chromatogram (TIC) of the propolis extracts from the negative mode analyzed by UHPLC/Q-TOF-MS. References [63,64,65] are cited in supplementary file.

## Abbreviations

DMEM: Dulbecco’s modified Eagle’s medium; CP, Chinese poplar propolis; FBS, fetal bovine serum; GLUT1, glucose transporter type 1; GLUT3, glucose transporter type 3; HK2, hexokinase 2; LDHA, lactate dehydrogenase A; PFK, phosphofructokinase; PKM2, pyruvate kinase muscle isozyme M2; ROS, reactive oxygen species; UHPLC/Q-TOF-MS, ultra-high-performance liquid chromatography/quadrupole time-of-flight mass spectrometry system; FBP, fructose 1,6-diphosphate; OXPHOS, oxidative phosphorylation; CAPE, caffeic acid phenethyl ester.
