# Supplementary material for: Integration with Transcriptomic and Metabolomic Analyses Reveals the In Vitro Cytotoxic Mechanisms of Chinese Poplar Propolis by Triggering the Glucose Metabolism in Human Hepatocellular Carcinoma Cells
Source: Nutrients. 2023 Oct 11;15(20):4329. doi: 10.3390/nu15204329 (PMC10610315; doi:10.3390/nu15204329)
Supplement: Supplementary file 1 [file nutrients-15-04329-s001.zip › nutrients-2636921-supplementary.pdf]

Supplemental Table S1. Sequences of the primers for qRT-PCR.

Genes	Primer sequence	Reference
GLUT1	F5'-GGCCAAGAGTGTGCTAAAGAA-3'<br>R5'-ACAGCGTTGATGCCAGACAG-3'	[63]
GLUT3	F5'-GGTGGCTGCTTTATGGGACT-3'<br>R5'-GTAAAACCCAGTAGCAGCGG-3'	This study
HK2	F5'-GAGCCACCACTCACCCTACT-3'<br>R5'-CCAGGCATTCGGCAATGTG-3'	[63]
PFK	F5'-AGGAGGGGAAGGGCATCT-3'<br>R5'-TTCCTATCAAATGGGGTTGG-3'	[64]
PKM2	F5'-ATGTCGAAGCCCCATAGTGAA-3'<br>R5'-TGGGTGGTGAATCAATGTCCA-3'	[63]
LDHA	F5'-ATGGCAACTCTAAAGGATCAGC-3'<br>R5'-CCAACCCCAACAACTGTAATCT-3'	[63]
β-actin	F5'-AGTTGCGTTACACCCTTTCTTG-3'<br>R5'-TCACCTTCACCGTTCCAGTTT-3'	[65]

Supplemental Table S2. TOP 100 components in ethanol extract of propolis by HPLC-QTOF-MC.

Compound Name	Retention Time	Ion Species
Mollugin	12.091	(M-H)-
Phenethyl caffeate	12.091	(M-H)-
Iso-sativan	12.091	(M-H)-
Homopterocarpin	12.091	(M-H)-
Glypallichalcone	12.091	(M-H)-
Batatasin I	12.091	(M-H)-
Cinnamyl benzoate	12.091	(M+HCOO)-
Benzyl cinnamate	12.091	(M+HCOO)-
Prenyl caffeate	11.558	(M-H)-
Tremetone	11.558	(M+HCOO)-
Azaleatin	9.094	(M-H)-
Nodifloretin	9.094	(M-H)-
Rhamnetin	9.094	(M-H)-
Pedalitin	9.094	(M-H)-
Isorhamnetin	9.094	(M-H)-
Eupafolin	9.094	(M-H)-
Capillarisin	9.094	(M-H)-
Emodin	9.094	(M+HCOO)-
Aloeemodin	9.094	(M+HCOO)-
Apigenin	9.094	(M+HCOO)-
Baicalein	9.094	(M+HCOO)-
Galangin	9.094	(M+HCOO)-
Genistein	9.094	(M+HCOO)-
6-Hydroxyrubiadin	9.094	(M+HCOO)-
Thunberginol A	9.094	(M+HCOO)-
Sulfuretin	9.094	(M+HCOO)-
Rubilactone	9.094	(M+HCOO)-
Norwogonin	9.094	(M+HCOO)-
Morindon	9.094	(M+HCOO)-
Aurantio-obtusin	11.208	(M-H)-
Ombuin	11.208	(M-H)-
Jaceosidin	11.208	(M-H)-
Tricin	11.208	(M-H)-
Rhamnazin	11.208	(M-H)-
Cirsiliol	11.208	(M-H)-
Glycitein	11.208	(M+HCOO)-
Genkwanin	11.208	(M+HCOO)-
Izalpinin	11.208	(M+HCOO)-
Physcion	11.208	(M+HCOO)-
Calycosin	11.208	(M+HCOO)-
Oroxylin	11.208	(M+HCOO)-

Compound Name	Retention Time	Ion Species
Galangin 3-methyl ether	11.208	(M+HCOO)-
Damnacanthol	11.208	(M+HCOO)-
Cypripedin	11.208	(M+HCOO)-
Biochanin A	11.208	(M+HCOO)-
Acacetin	11.208	(M+HCOO)-
Xenognosin B	11.208	(M+HCOO)-
Wogonin	11.208	(M+HCOO)-
Stevein	11.208	(M+HCOO)-
Questin	11.208	(M+HCOO)-
Prunetin	11.208	(M+HCOO)-
Obtusifolin	11.208	(M+HCOO)-
Alpinone	9.044	(M-H)-
Pabulenol	9.044	(M-H)-
Brasilin	9.044	(M-H)-
Heraclenin	9.044	(M-H)-
Isogosferol	9.044	(M-H)-
Isooxypeucedanin	9.044	(M-H)-
Kushenin	9.044	(M-H)-
Moracin B	9.044	(M-H)-
Oxypeucedanin	9.044	(M-H)-
Phyllodulcin	9.044	(M-H)-
Quinquangulin	9.044	(M-H)-
Sakuranetin	9.044	(M-H)-
Vestitone	9.044	(M-H)-
5,7,4’-Trihydroxy-8-methylflavanone	9.044	(M-H)-
Thespone	9.044	(M+HCOO)-
Chrysarobin	9.044	(M+HCOO)-
Flavidin	9.044	(M+HCOO)-
Moscatin	9.044	(M+HCOO)-
(Z,E)-2-(3,5-Dihydroxyphenyl) ethenyl ester of 3-(3,4-dihydroxyphenyl)-2-propenoic acid	12.157	(M-H)-
1,7-Dihydroxy-3,9-dimethoxy pterocarpene	12.157	(M-H)-
(-)-3-Hydroxy-4-methoxy-8-9-methylenedioxy pterocarpan	12.157	(M-H)-
Kumatakenin	12.157	(M-H)-
7-Methoxy-aromadendrin	12.157	(M-H)-
Pectolinarigenin	12.157	(M-H)-
Panicolin	12.157	(M-H)-
Persicogenin	12.157	(M-H)-
Pisatin	12.157	(M-H)-
Rhynchotechol	12.157	(M-H)-

Compound Name	Retention Time	Ion Species
trans-3-Acetoxy-5,7-dihydroxyflavanone	12.157	(M-H)-
Cirsimaritin	12.157	(M-H)-
5,8-Dihydroxy-6,7-dimethoxyflavone	12.157	(M-H)-
2',7-Dihydroxy-4',5'-dimethoxyisoflavone	12.157	(M-H)-
(Z,E)-2-(3,4-Dihydroxyphenyl) ethenyl ester of 3-(3,4-dihydroxyphenyl)-2-propenoic acid	12.157	(M-H)-
Tectochrysin	12.157	(M+HCOO)-
Dalbergin	12.157	(M+HCOO)-
Formononetin	12.157	(M+HCOO)-
Isodalbergin	12.157	(M+HCOO)-
7-Methoxy-4'-hydroxyflavone	12.157	(M+HCOO)-
Pallidiflorin	12.157	(M+HCOO)-
Pratol	12.157	(M+HCOO)-
Rubiadin-1-methyl ether	12.157	(M+HCOO)-
Medicarpin	11.658	(M-H)-
Echinatin	11.658	(M-H)-
Vignafuran	11.658	(M-H)-
Isoimperatorin	11.658	(M-H)-
Imperatorin	11.658	(M-H)-
Cardamonin	11.658	(M-H)-

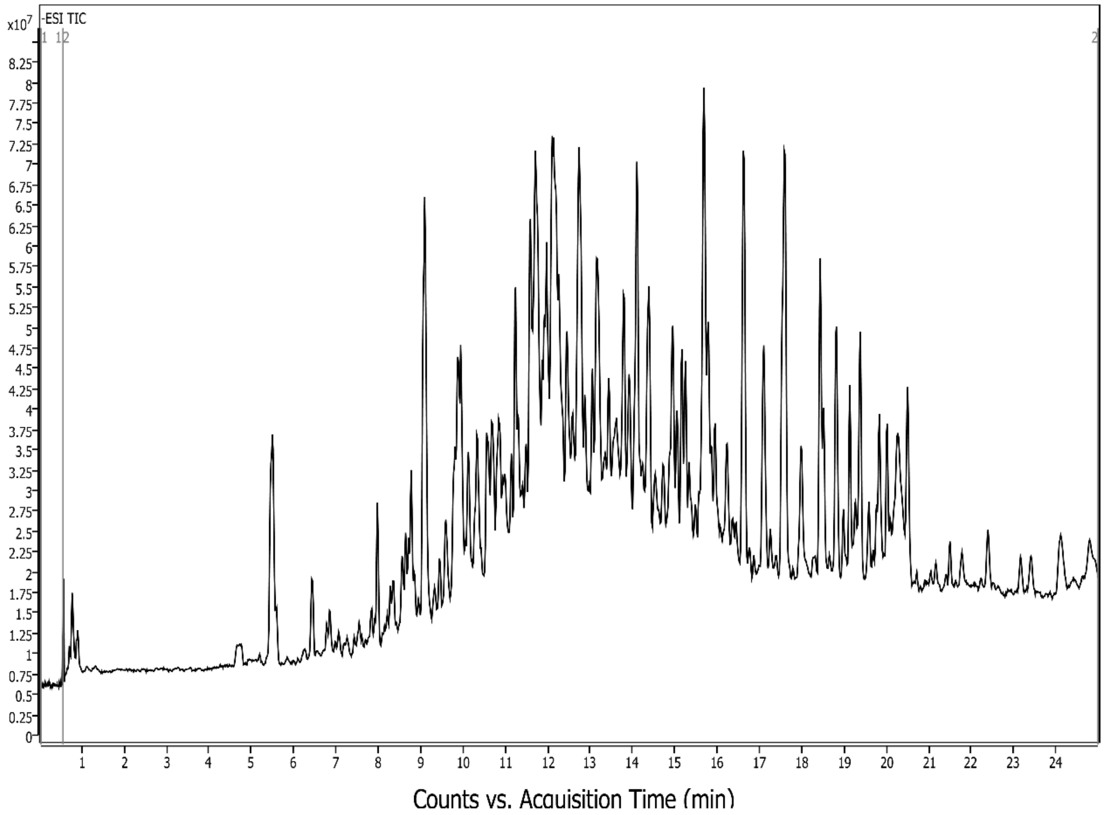

Figure S1. Total ion current chromatogram (TIC) of the propolis extracts from the negative mode analyzed by UHPLC/Q-TOF-MS.
